# Long-term mesh erosion rate following abdominal robotic reconstructive pelvic floor surgery: a prospective study and overview of the literature

**DOI:** 10.1007/s00192-019-03990-1

**Published:** 2019-06-20

**Authors:** Femke van Zanten, Jan J. van Iersel, Tim J. C. Paulides, Paul M. Verheijen, Ivo A. M. J. Broeders, Esther C. J. Consten, Egbert Lenters, Steven E. Schraffordt Koops

**Affiliations:** 1 Department of Gynecology, Meander Medical Center, Maatweg 3, 3813 TZ Amersfoort, The Netherlands; 2 Faculty of Electrical Engineering, Mathematics & Computer Science, Twente University, Enschede, The Netherlands; 3 Department of Surgery, Meander Medical Center, Amersfoort, The Netherlands; 4 Department of Surgery, University Medical Center Groningen, Groningen, The Netherlands

**Keywords:** Erosion, Mesh exposure, Pelvic organ prolapse, Robotic, Sacrocolpopexy, Sacrocolporectopexy

## Abstract

**Introduction and hypothesis:**

The use of synthetic mesh in transvaginal pelvic floor surgery has been subject to debate internationally. Although mesh erosion appears to be less associated with an abdominal approach, the long-term outcome has not been studied intensively. This study was set up to determine the long-term mesh erosion rate following abdominal pelvic reconstructive surgery.

**Methods:**

A prospective, observational cohort study was conducted in a tertiary care setting. All consecutive female patients who underwent robot-assisted laparoscopic sacrocolpopexy and sacrocolporectopexy in 2011 and 2012 were included. Primary outcome was mesh erosion. Preoperative and postoperative evaluation (6 weeks, 1 year, 5 years) with a clinical examination and questionnaire regarding pelvic floor symptoms was performed. Mesh-related complications were assessed using a transparent vaginal speculum, proctoscopy, and digital vaginal and rectal examination. Kaplan–Meier estimates were calculated for mesh erosion. A review of the literature on mesh exposure after minimally invasive sacrocolpopexy was performed (≥12 months’ follow-up).

**Results:**

Ninety-six of the 130 patients included (73.8%) were clinically examined. Median follow-up time was 48.1 months (range 36.0–62.1). Three mesh erosions were diagnosed (3.1%; Kaplan–Meier 4.9%, 95% confidence interval 0–11.0): one bladder erosion for which mesh resection and an omental patch interposition were performed, and two asymptomatic vaginal erosions (at 42.7 and 42.3 months) treated with estrogen cream in one. Additionally, 22 patients responded solely by questionnaire and/or telephone; none reported mesh-related complaints. The literature, mostly based on retrospective studies, described a median mesh erosion rate of 1.9% (range 0–13.3%).

**Conclusions:**

The long-term rate of mesh erosion following an abdominally placed synthetic graft is low.

**Electronic supplementary material:**

The online version of this article (10.1007/s00192-019-03990-1) contains supplementary material, which is available to authorized users

## Introduction

The use of synthetic mesh in pelvic floor surgery has been subject to debate. In 2008 and 2011, the US Food and Drug Administration (FDA) warned about the high rate of mesh-related complications following transvaginal pelvic organ prolapse repair [1]. The FDA warnings were underlined by a systematic review reporting an incidence of mesh erosion of 10.3% (range 0–29.7%, *n* = 11.785) following transvaginal pelvic organ prolapse repair in the first postoperative year [2]. Recent literature on transvaginal repair has confirmed this high incidence [3]. Transabdominal approaches for pelvic reconstructive surgery are associated with a much lower incidence of mesh erosion [1, 4]. However, most studies describing mesh erosion are retrospective with short-term follow-up. Research focusing specifically on long-term mesh-related morbidity is lacking.

Minimally invasive sacrocolpopexy is currently the preferred treatment for apical prolapse, and ventral mesh rectopexy has gained increasing worldwide acceptance for rectal prolapse [5, 6]. More recently, the two abdominal procedures combined have been described and are being used as a treatment for combined pathology [7, 8].

It is against this backdrop that we designed a study to evaluate the long-term mesh erosion rate following robot-assisted laparoscopic sacrocolpopexy (RSC) and robot-assisted laparoscopic sacrocolporectopexy (RSCR). Second, we performed a literature review on mesh erosion after minimally invasive sacrocolpopexy with a minimum follow-up duration of 12 months.

## Materials and methods

### Study design and participants

All consecutive female patients who underwent RSC or RSCR at a tertiary referral center for pelvic floor disorders in the Netherlands in 2011 and 2012 were prospectively included. The set-up was an observational cohort study. The primary outcome was long-term mesh erosion.

### Inclusion and exclusion criteria

Inclusion criteria were patients with symptomatic vaginal vault prolapse (simplified pelvic organ prolapse quantification [simplified POP-Q] stage ≥2) and patients with additional symptomatic internal/external rectal prolapse (Oxford Grading System grade ≥ 3; an additional enterocele or rectocele may be present). Exclusion criteria were conversion to another procedure without mesh usage, poor health status with inability to undergo general anesthesia, patients younger than 18 years, ≥3 previous laparotomic surgeries, planned pregnancy, known pelvic/abdominal malignancies. This study was carried out in accordance with the ethical standards of the Central Committee on Research Involving Human Subjects and with the Declaration of Helsinki. Patients gave informed consent before inclusion. 

### Clinical evaluation

Patients were clinically reviewed preoperatively and postoperatively at 6 weeks, 1 year, 5 years and in cases where complaints occurred. Rectal prolapse was diagnosed and evaluated at follow-up using the Oxford Grading System by proctoscopy and dynamic MRI [9]. The simplified POP-Q was used to determine vaginal prolapse [10]. At follow-up, all patients underwent a digital vaginal and rectal examination, a proctoscopy, and a vaginal speculum examination to assess mesh-related complications. Both proctoscope and speculum were transparent. Patients were examined in the supine lithotomy position using leg supports, both in rest and during maximal Valsalva. Clinical examination was performed by an objective researcher (not blinded). If mesh-related morbidity was suspected, a second examination by a gynecologist was performed to confirm the diagnosis. Mesh erosion was graded according to the International Urogynaecological Association (IUGA) and the International Continence Society (ICS) joint terminology and category, time, and site (CTS) classification, although we used the term mesh erosion instead of mesh exposure [11]. During every evaluation (pre- and postoperatively), patients received a surgical and urogynecological questionnaire on paper, which included questions regarding symptoms of bulge, micturition (Urinary Distress Inventory; UDI-6), defecation (obstructive defecation and fecal incontinence), and quality of life (Pelvic Floor Impact Questionnaire; PFIQ-7) [12, 13]. In case patients declined clinical evaluation, patients were invited to return the questionnaire by post. Questions regarding mesh-related morbidity were asked postoperatively during the clinical evaluation or, if patients declined examination, by telephone: “vaginal/rectal bleeding or discharge,” “vaginal/rectal pain,” “pelvic pain (either spontaneous or during physical activity),” “recurrent urinary tract infection.” Patients were considered lost to follow-up in cases where no physical examination or no questionnaire was available. Solely patients with a postoperative physical examination available were included in the analysis to determine the mesh erosion rate.

### Surgical technique

All procedures were performed using the da Vinci robot (Intuitive Surgical, Sunnyvale, CA, USA) by three colon surgeons and two urogynecologists with ≥10 years’ experience. Every patient received prophylactic intravenous antibiotics (1,000 mg cefazolin and 500 mg metronidazole) 15 min prior to incision. The RSC procedures, with or without supracervical hysterectomy, were performed according to the technique described by Clifton et al. [14]. RSC was performed solely by the gynecologist. The technique of RSCR was performed jointly by one colorectal and one urogynecological surgeon. The technique of RSCR has been previously described by our study group [7]. All meshes (type 1, macroporous polypropylene, Prolene, Ethicon Inc., Johnson & Johnson, Hamburg, Germany, weight 80–85 g/m^2^) were distally attached using non-absorbable sutures (Ethibond; Ethicon, Johnson & Johnson, Hamburg, Germany) and anchored proximally to the sacral promontory using titanium tacks (Autosuture Protack 5 mm; Covidien, USA). Two meshes were used, configured into a “Y” shape intracorporeally. The peritoneum was approximated to cover the implant using a 23-cm V-Loc suture (Covidien, Mansfield, MA, USA). The surgeon performed a vaginal/rectal examination at the end of each procedure to exclude a possible (suture) perforation of the vaginal and/or rectal wall and to determine the correct position of the mesh.

### Review of the literature

To compare our mesh erosion rate with the literature, a literature search was performed describing mesh erosion after minimally invasive sacrocolpopexy with a minimal duration of follow-up of 12 months (Appendix A). Studies describing mesh erosion after open/minimally invasive sacrocolporectopexy were described separately (Appendix A).

### Statistical analysis

Statistical Package for the Social Sciences, version 20.0 (IBM, Armonk, NY, USA) was used for statistical analysis. Data are presented as percentage, mean ± standard deviation (SD), and median and range. Chi-squared test, Fisher’s exact test and independent sample *t* test were used to compare variables between groups for categorical and continuous data. Because of differences in follow-up between patients, the Kaplan–Meier method with 95% confidence intervals was used to estimate the erosion rate at various time points. The log-rank test was used to compare Kaplan–Meier estimates between subgroups.

## Results

### Patients

**Fig. 1:**
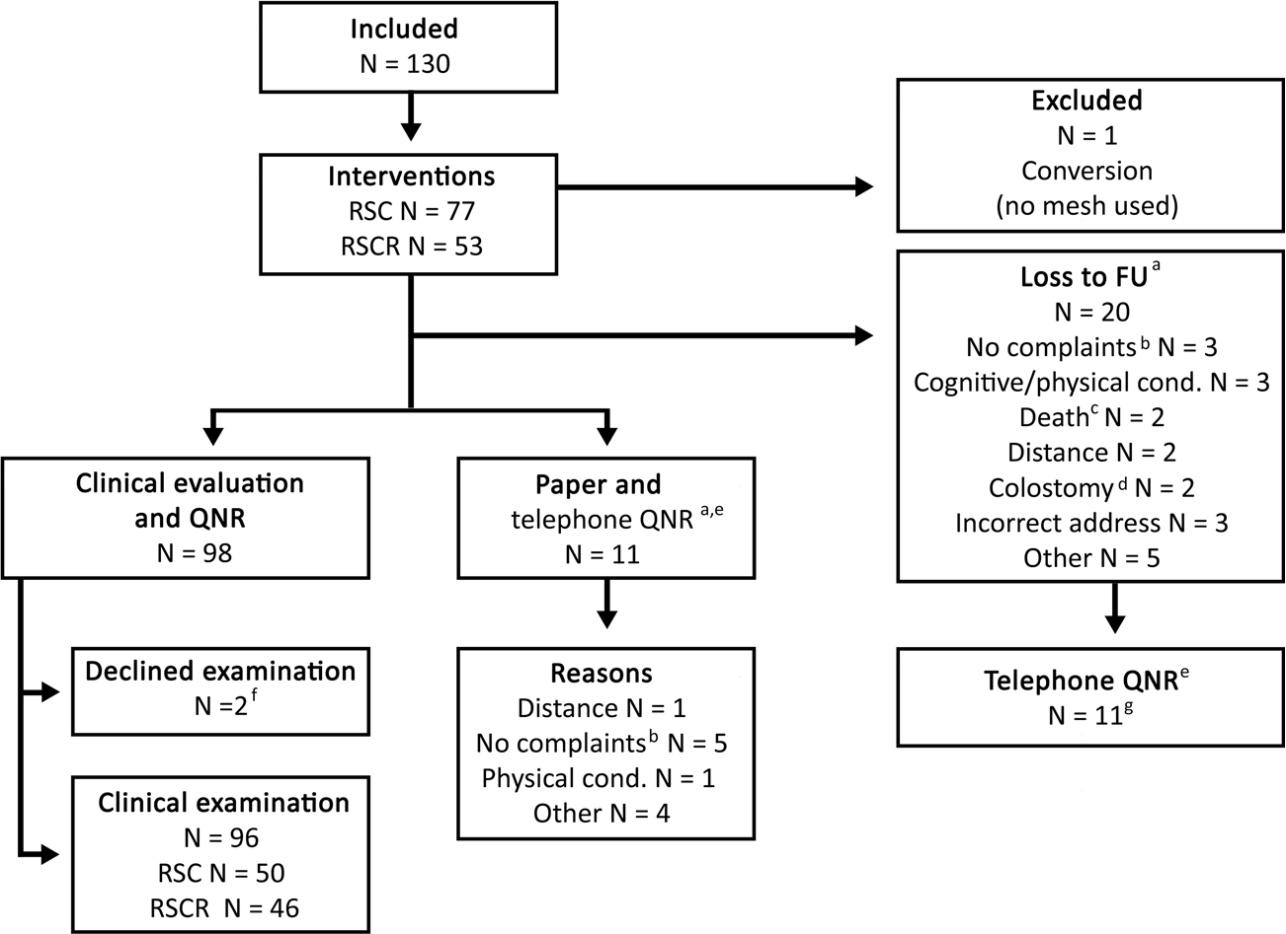
Flowchart of patients included. ^a^The general practitioner was contacted in the case of incorrect address details. ^b^Patients had no complaints and patients themselves judged an examination to be unnecessary. ^c^Due to natural causes. ^d^Two patients received a colostomy and declined further participation: one because of therapy-resistant fecal incontinence and extensive sphincter dysfunction, and one because of disabling obstructed defecation. ^e^Questionnaires regarding pelvic floor symptoms. The paper questionnaire was discussed during consultation. Patients who were unable to attend or declined clinical examination were asked to send back the questionnaire by post. These patients and patients who were lost to follow-up were contacted additionally by telephone to ask for specific anamnestic mesh-related morbidity. ^f^No anamnestic mesh-related complaints. ^g^Patients who could not be reached: death (due to natural causes) *n* = 2, cognitive/physical condition *n* = 3, untraceable *n* = 3, other *n* = 1. cond. condition *FU* follow-up, *RSC* robot-assisted laparoscopic sacrocolpopexy, *RSCR* robot-assisted laparoscopic sacrocolporectopexy, *QNR* questionnaire

One hundred and thirty patients underwent surgery. One procedure (0.8%) was converted to vaginal prolapse surgery owing to a pre-sacral bleeding. This patient was excluded from the study, since no mesh was placed. Twenty patients (15.4%) were lost to follow-up and 11 patients (8.5%) solely responded by questionnaire. In total, 96 patients (73.8%) were physically examined in the outpatient clinic. The flow chart of patients included is shown in Fig. 1.

### Demographics and operative data

Fifty women (52.1%) underwent an RSC and 46 women (47.9%) an RSCR (Table 1). RSC and RSCR were combined with a concomitant supracervical hysterectomy in 36 (72%) and in 25 (54.3%) cases respectively. Mean age and body mass index of all patients were 62.3 ± 10.4 years and 26.1 ± 4.2 kg/m^2^ respectively. Two cases (2.1%) were converted to an open procedure (extensive intra-abdominal adhesions *n* = 1; anesthesia-related issues *n* = 1). Intra-operative complications occurred in 3 (3.1%) patients; 2 small bladder perforations in the bladder dome and 1 minor serosal small bowel lesion. No (mesh-related) postoperative complications were observed in these specific patients. Median follow-up time was 48.1 months (range 36.0–62.1).

**Table 1:** Patient demographics and operative data

	Total (*N* = 96)	RSC (*n* = 50)^a^	RSCR (*n* = 46)	*p* value
Mean age (SD)	62.3 (10.4)	62.4 (9.5)	62.2 (11.5)	0.922
Mean ASA classification (SD)	1.8 (0.5)	1.7 (0.5)	1.9 (0.5)	0.112
Mean parity (SD)	2.8 (1.0)	2.8 (1.1)	2.8 (1.0)	0.898
Mean BMI (SD)	26.1 (4.2)	25.9 (3.7)	26.3 (4.7)	0.683
Episiotomy (%)	51 (53.1)	29 (58.0)	22 (47.8)	0.318
Prolapse first degree relative (%)	35 (36.5)	20 (40.0)	15 (32.6)	0.648
Smoking (%)	23 (24.0)	12 (24.0)	11 (23.9)	0.957
Sexually active (%)	45 (46.9)	25 (50.0)	20 (43.5)	0.198
History (%)				
TVT	5 (5.2)	1 (2.0)	4 (8.7)	0.195^b^
Burch colposuspension	1 (1.0)	1 (2.0)	0	1.000^b^
Hysterectomy	34 (35.4)	14 (28.0)	20 (43.5)	0.113
Sacrocolpopexy	1 (1.0)	1 (2.0)	0	1.000^b^
Anterior colporrhaphy	20 (20.8)	9 (18.0)	11 (23.9)	0.476
Posterior colporrhaphy	19 (19.8)	8 (16.0)	11 (23.9)	0.331
Rectopexy	2 (2.1)	1 (2.0)	1 (2.2)	1.000^b^
Perineal procedure	2 (2.1)	0	2 (4.3)	0.227^b^
Sphincter procedure	0	0	0	N/A
Hemorrhoidectomy	2 (2.1)	0	2 (4.3)	0.227^b^
Other abdominal surgery	32 (33.3)	15 (30.0)	17 (37.0)	0.470
Rectal prolapse (%)				
ERP	4 (4.2)	0	4 (8.7)	0.049^b^
IRP or/and symptomatic rectocele	49 (51.0)	21 (42.0)	28 (60.9)	0.065
with enterocele	15 (15.6)	3 (6.0)	12 (26.1)	0.007
Simplified POP-Q, mean (SD)				
POP-Q Ba	2.4 (1.0)	2.6 (0.9)	2.4 (0.9)	0.947
POP-Q Bp	1.9 (1.0)	1.9 (1.0)	2.2 (1.0)	0.149
POP-Q C	2.5 (1.0)	2.9 (0.9)	2.3 (1.0)	0.021
POP-Q D	2.0 (1.0)	2.4 (1.0)	2.2 (1.0)	0.273
Concomitant supracervical hysterectomy (%)	61 (63.5)	36 (72.0)	25 (54.3)	0.073
Conversion (%)	2 (2.1)	1 (2.0)	1 (2.2)	1.000^b^
Intra-operative complications (%)	3 (3.1)	0	3 (6.5)	0.106
Mean LOS, nights (SD)	2.8 (1.2)	2.3 (0.9)	3.4 (1.2)	<0.0005
Early postoperative complications (%)				
CD grade ≤ 2	2 (2.1)	2 (4.0)	0	0.496^b^
CD grade ≥ 3	1 (1.0)	1 (2.0)	0	1.000^b^
Mesh erosion (%)	3 (3.1)	2 (4.0)	1 (2.2)	1.000^b^
Postoperative in-hospital mortality (%)	0	0	0	N/A

*RSC* robot-assisted laparoscopic sacrocolpopexy, *RSCR* robot-assisted laparoscopic sacrocolporectopexy, *simplified POP-Q* simplified pelvic organ prolapse quantification, *SD* standard deviation, *ASA* American Society of Anesthesiologists, *BMI* body mass index, *TVT* tension-free vaginal tape, *N/A* not applicable, *ERP* external rectal prolapse, *IRP* internal rectal prolapse, symptomatic, LOS length of hospital stay, *CD* Clavien–Dindo classification

^a^Two RSCs were combined with a TVT

^b^Fisher’s exact test

### Mesh-related complications

**Fig. 2:**
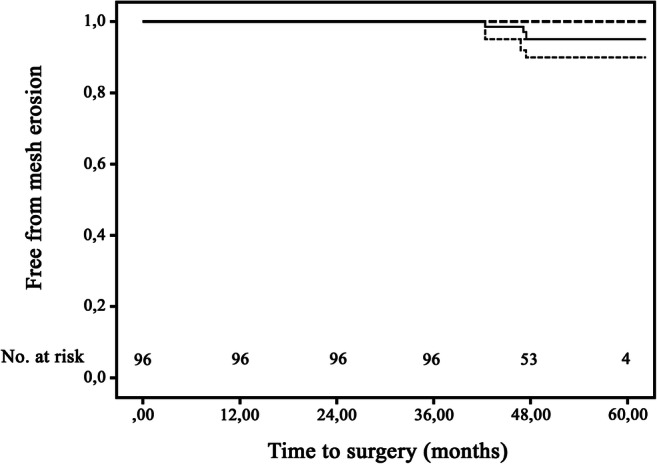
Kaplan–Meier curve of mesh erosion. Kaplan–Meier curve for mesh erosion after RSC and RSCR (*straight line*). *Dotted gray lines* represent upper and lower 95% confidence intervals. The duration of event-free survival was measured from the date of surgery to the time of the event (complete) or the last follow-up (censored).

Three patients (3.1%) developed mesh erosion during follow-up (Table 2). The accompanying actuarial erosion rates for the total cohort were 0% after 1 year, 0% after 3 years, and 4.9% after 5 years (95% confidence interval 0–11.0; Fig. 2; Kaplan–Meier curve). The Kaplan–Meier estimates for RSC and RSCR after 5 years were 5.3 (95% CI 0–12.4%) and 3.0 (95% CI 0–8.9) respectively. No significant difference between the two subgroups could be found (*p* = 0.808). The first patient presented with pain, dysfunctional voiding, and recurrent urinary tract infections 45 months after RSC with supracervical hysterectomy. A small defect of the posterior wall of the bladder with mesh exposure was observed with cystoscopy. The mesh was removed and an omental patch interposition was performed. The second mesh erosion was discovered during regular follow-up 42.7 months following RSCR with supracervical hysterectomy. An asymptomatic erosion was found in the posterior wall of the vagina for which vaginal estrogen was prescribed. The third mesh erosion was also asymptomatic and was found in the posterior wall of the vagina at 42.3 months after RSC. Since the mesh exposure was so small, expectant management was chosen. All three patients who developed a mesh erosion had an extensive surgical pelvic floor history (Table 2). Two of the three women were postmenopausal. The three patients with mesh erosion had some of the characteristics and recognized risk factors for mesh erosion, including history of pelvic floor surgery (*n* = 3), vaginal atrophy (*n* = 3), smoking (*n* = 1), sexual activity (*n* = 1), and age > 70 years (*n* = 2). During the intraoperative vaginal examination of one of these patients, a perforating suture was removed, which may be another risk factor for the occurrence of mesh erosion.

**Table 2:** Mesh erosions in the current study

Age, years (ASA)	Surgical history	Procedure	Location, symptoms	CTS [11]	Defect (cm)	Examination, months	Treatment
50 (2)	Cervical amputation, ventral mesh rectopexy, anterior and posterior Colporrhaphy	RSC with supracervical hysterectomy	Bladder, posterior wall, symptomatic	4B/T4/S3	< 1	45.0	Mesh resection and omental patch interposition
77 (2)	Unknown prolapse surgery, anterior and posterior colporrhaphy	RSCR with supracervical hysterectomy	Vagina, posterior wall, asymptomatic	2A/T4/S1	1	42.7	Vaginal estrogen therapy twice a week
74 (2)	Hysterectomy, posterior colporrhaphy and McCall	RSC	Vagina, posterior wall, asymptomatic	2A/T4/S1	< 1	42.3	Expectant management

*ASA* American Society of Anesthesiologists *CTS* category (C), time (T) and site (S)

Four (4.2%) other post-menopausal patients (mean age 70.3 ± 7.8 years), all with vaginal atrophy, experienced vaginal discomfort examining the distal side of the mesh. No mesh erosion or other mucosal abnormalities were observed. One of these patients developed postoperative new-onset dyspareunia, but declined the use of vaginal estrogens, because of the sporadic occurrence of complaints. All four patients were offered vaginal estrogen therapy, only two (both sexually active) patients accepted the prescription. No other mesh-related morbidity was observed in the complete cohort.

Twenty-two patients were assessed solely by questionnaire, none reported mesh-related complaints.

### Overview of literature

Details of the literature search and a flow-chart of studies included are presented in Appendix A. Sixty-five studies described mesh erosion after laparoscopic sacrocolpopexy (LSC) and/or RSC (Table 3). Most studies were of a retrospective design (73.8%). The literature on LSC and RSC shows erosion rates between 0 and 13.3% (range of number of patients included: 12–4,552; range of follow-up 12–72 months) [3, 5, 15–77]. The articles that were included differed in their methods and inclusion criteria. Some studies solely included posthysterectomy patients. Other studies also included patients with their uterus still present, performing either a total hysterectomy or supracervical hysterectomy. Furthermore, different types of mesh were used throughout the studies. Eighty-three percent of the articles reported an erosion percentage of ≤5% with an overall median erosion rate of 1.9%. Six studies (9.2%) had a follow-up duration of more than 48 months [24, 34, 43, 51, 61, 74]. One of these six studies included 391 patients. The authors reported mesh-related complications requiring surgical intervention in 2.8% [51]. However, follow-up in this study, was carried out by telephone interview and the numbers could therefore be underestimated. The other five studies reported on 361 patients in total, with 15 mesh erosions (4.1%; range of mesh erosion 2.9–7.8%). This is in line with the mesh erosion rate found in our study. Dandolu et al. [3] described a large retrospective cohort of patients (*N* = 4,552, follow-up ≥2 years) with an apical prolapse who underwent LSC. Mesh removal or revision occurred in 52 patients (1.7%). One study by Geller et al. [59] reported a mesh erosion rate of 13%. The study included solely 15 patients, which could possibly explain the high erosion rate. Practically all erosions reported in the literature were symptomatic. One study mentions asymptomatic mesh erosion [62]. Three studies on sacrocolpopexy using a light-weight mesh show an erosion percentage of 0% after 12 months of follow-up [41, 68, 71].

**Table 3:** Mesh erosion following minimally invasive sacrocolpopexy with synthetic mesh (≥ 12 months of follow-up)

Reference	Number of patients	Material and type of mesh	Vaginal/ rectal examination mesh	Follow-up, months (median)	Mesh complication (%)	Mesh erosion (%)
Laparoscopic and robotic						
Paraiso et al. [5]	33 L, 35 R	PP, 1	Only vaginal	12	0 L, 2^f^ (5.7) R	0 L, 2^f^ (5.7) R
Chan et al. [15]	20 L, 16 R	PP, 1	Only vaginal	39 L, 16 R	0 L, 0 R	0 L, 0 R
Tan-Kim et al. [16]	58 L, 41 R	PP, 1	Only vaginal	12 L, 19 R	2 (3.6) L, 2 (4.9) R	2 (3.6) L, 2 (4.9) R
Seror et al. [17]	47 L, 20 R	PP, 1	Only vaginal	18 L, 15 R	1 (2.1) L, 0 R	1 (2.1) L, 0 R
Joubert et al. [18]	39 L, 17 R	PP, 1/PE, 3	Only vaginal	14.9 L, 12 R	2 (5.1) L, 0 R	2 (5.1) L, 0 *R*
Tan-Kim et al. [19]	32 L, 32 R	PP, 1	Only vaginal	12	1 (3.1) L, 2 (6.3) R	1 (3.1) L, 1 (3.1) R
Kenton et al. [20]	33 L, 33R	PP, 1	Only vaginal	12	0	0
Laparoscopic						
Antiphon et al. [21]	104	PE, 3	Only vaginal	17	2 (1.9)	0
Gadonneix et al. [22]	46	PE, 3	Only vaginal	24	0	0
Paraiso et al. [23]	56	PP, 1	n/d	13.5^d^	2 (3.6)	2 (3.6)
Ross and Preston [24]	51	PP, 1	Only vaginal	60	6 (11.8)	4 (7.8)
Rozet et al. [25]	325	PE, 3	Only vaginal	14.5^d^	8^g^ (2.5)	3 (0.9)
Agarwala et al. [26]	72	PP, 1	Only vaginal	24	1 (1.4)	0
Rivoire et al. [27]	108	PP, 1	Only vaginal	33.7^d^	9 (8.3)	7 (6.5)
Stepanian et al. [28]	402	PP, 1	n/d	12	12 (3.0)	5 (1.2)
Deprest et al. [29]	104^a^	PP, 1^a^	Only vaginal	33^d^	12 (11.5)	8 (7.7)^i^
Granese et al. [30]	165	PP, 1	Yes, both	43	7 (4.2)^h^	1 (0.6)
Loffeld et al. [31]	20	PP, 1	Only vaginal	45^d^	1 (5.0)	1 (5.0)
North et al. [32]	22	PP, 1	Only vaginal	27.5^d^	1 (4.5)	1 (4.5)
Akladios et al. [33]	48	PP, 1	Only vaginal	15.8^d^	1 (2.2)	1 (2.2)
Sabbagh et al. [34]	132	PP, 1	Only vaginal	60	6 (4.5)	5 (3.8)
Maher et al. [35]	53	PP, 1	Only vaginal	24^d^	1 (1.9)	1 (1.9)
Sergent et al. [36]	116	PE, 3	Only vaginal	34.2	5 (4.3)	4 (3.4)
Perez et al. [37]	85	PE, 3	Only vaginal	12	5 (5.9)	3 (3.5)
Price et al. [38]	84	PP, 1	Only vaginal	24^d^	5 (6.0)	5 (6.0)^j^
Freeman et al. [39]	23	PP, 1	Only vaginal	12	0	0
Leruth et al. [40]	55	PE, 3	Only vaginal	25^d^	0	0
Liu et al. [41]	39	PP, 1	Only vaginal	12	0	0
Park et al. [42]	54	PP, 1	Only vaginal	29.7^d^	3 (5.6)	3 (5.6)
Sarlos et al. [43]	68	PP, 1	Only vaginal	60^d^	2 (2.9)	2 (2.9)
El Hamamsy and Fayyad [44]	220	PP, 1	Only vaginal	12	2 (0.9)	2 (0.9)
Estrade et al. [45]	35	PE, 3	Only vaginal	13.2	1 (2.9)	1 (2.9)
Gracia et al. [46]	30	PP, 1	Only vaginal	12	0	0
Vieillefosse et al. [47]	100	PP, 1/PE, 3	Only vaginal	23.6	2 (2.0)	2 (2.0)
Costantini et al. [48]	60	PP, 1	Only vaginal	41.7^d^	3 (5.0)	3 (5.0)
Dandolu et al. [3]	4,552	n/d	n/a	24	52 (1.7)	52 (1.7)
Liang et al. [49]	30	PP, 1	Only vaginal	36	3 (10)	3 (10)
Lizee et al. [50]	60	PE, 3	Only vaginal	27	1 (1.7)	1 (1.7)
Vandendriessche et al. [51]	391^b^	PP, 1/PE, 3	No, telephone FU	53.3	11 (2.8)	7 (1.8)
Zebede et al. [52]	144	PP, 1	Only vaginal	21	4 (2.8)	0
Pan et al. [53]	99	PP, 1	Only vaginal	33^d^	0	0
Chen and Hua [54]	102	PP, 1	Only vaginal	24	1 (1.0)	1 (1.0)
Robotic						
Elliott et al. [55]	42	PP, 1	Only vaginal	36^d^	3 (7.1)	2 (4.8)
Benson et al. [56]	33	PP, 1	n/d	20.7–38.4^e^	2 (6.1)	0
Shveiky et al. [57]	17	PP, 1	Only vaginal	12.3	0	0
Xylinas et al. [58]	12	PP, 1	n/d	19.1	0	0
Geller et al. [59]	15	PP, 1	Only vaginal	14.8^d^	2 (13.3)	2 (13.3)
Moreno Sierra et al. [60]	31	PP, 1	Only vaginal	24.5^d^	1 (3.2)	0
Shimko et al. [61]	40	PP, 1	Only vaginal	62	2 (5.0)	2 (5.0)
Geller et al. [62]	23	PP, 1	Only vaginal	44.2^d^	2 (8.7)	2 (8.7)
Göçmen et al. [63]	12	PP, 1	n/d	12	0	0
Mourik et al. [64]	50^c^	PP, 1	Only vaginal	16	1 (2.0)	0
Siddiqui et al. [65]	70	PP, 1	Only vaginal	18.3^d^	3 (4.3)	3 (4.3)
Belsante et al. [66]	35	PP, 1	Only vaginal	28	1 (2.9)	1 (2.9)
Louis-Sylvestre and Herry [67]	90	PE, 3	n/d	15.6^d^	1 (1.1)	1 (1.1)
Salamon et al. [68]	118	PP, 1	Only vaginal	12	0	0
Barboglio et al. [69]	127	PP, 1	Only vaginal	12	3 (2.4)	3 (2.4)
Borahay et al. [70]	20	PP, 1	Only vaginal	17.3^d^	0	0
Culligan et al. [71]	143	PP, 1	Only vaginal	12	0	0
Ploumidis et al. [72]	95	PP, 1	Only vaginal	34.8	1 (1.1)	1 (1.1)
Jambusaria et al. [73]	30	PP, 1	Only vaginal	12	1 (3.3)	1 (3.3)
Linder et al. [74]	70	PP, 1	n/d	72	2 (2.9)	2 (2.9)
Myers et al. [75]	83	PP, 1	Only vaginal	12.8	4 (4.8)	4 (4.8)
Prendergast et al. [76]	33	PP, 1	Only vaginal	12	2 (6.1)	2 (6.1)
Linder et al. [77]	132	PP, 1	Only vaginal	33	8 (6.1)	8 (6.1)

*L* laparoscopic, *R* robot, *PP* polypropylene, *PE* polyester, *n/d* not described, *n/a* not applicable, *FU* follow-up

^a^39 with porcine dermis, 65 with PP

^b^Long-term follow-up performed with telephone/postal questionnaire

^c^All procedures were robot-assisted laparoscopic sacrohysteropexy

^d^Mean instead of median

^e^Patients with laparoscopic sacrocolpopexy: mean FU 38.4 months, patients with laparoscopic sacrocolpopexy and hysterectomy: mean FU 20.7

^f^One erosion was from a tension-free vaginal tape

^g^Two patients with an additional tension-free vaginal tape had urinary retention requiring section of the tape

^h^Includes detachment of the mesh

^i^Two after sacrocolpopexy with xenograft, 6 after sacrocolpopexy with PP

^j^Four out of 5 were suture erosions

Four studies described mesh erosion after open/minimal invasive sacrocolporectopexy, varying from 2.0 to 5.4% (median range of follow-up 195 days to 64 months) [7, 8, 78, 79]. Only 1 of the 4 studies performed a rectal and vaginal examination after 12 months of follow-up and noted a 2% erosion rate [7].

## Discussion

Synthetic meshes have been used in pelvic reconstructive surgery to reinforce weak or defective supportive tissue since 1959 [80]. The use of synthetic mesh potentially adds to the complication profile and mesh-related morbidity can have a considerable impact on the quality of life [81]. The introduction of transvaginal procedures showed a high risk of mesh-related complications [1]. This study with long-term follow-up shows that mesh-related morbidity following a minimally invasive abdominal pelvic floor repair is low.

In total, there were 3 patients with a mesh erosion (3.1%), of which 2 were asymptomatic. Two of these 3 patients underwent a concomitant supracervical hysterectomy. A total hysterectomy is associated with a four times higher risk of mesh erosion compared with sacrocolpopexy without hysterectomy [82]. A subtotal hysterectomy, however, appears to generate mesh erosion rates comparable with patients with a history of a hysterectomy undergoing a sacrocolpopexy [82]. Other known predictors of mesh erosion include the use of steroids, diabetes, level of surgeon experience, intra-abdominal adhesions, and postoperative pelvic hematoma [3, 81–84].

In this study, a monofilament and macroporous (>75 μm, type I) mesh was used, allowing host cell colonization with collagen deposition, angiogenesis, and infiltration of leukocytes, resulting in good support and a reduced risk of infection [28]. Research showed that synthetic meshes with smaller pores (type II and III) are associated with a higher erosion rate [81, 85]. It has been suggested that lightweight meshes might be less prone to erosion, but may have a higher recurrence rate than heavy-weight grafts. Three studies show a 0% mesh erosion rate one year after the use of light-weight mesh [41, 68, 71]. Studies with longer follow-up or comparative studies for an abdominal prolapse repair, however, do not exist. Data on mesh usage with abdominal hernia repairs suggests an impact of the weight of the mesh, but the optimal balance between weight and porosity is unknown [86]. No significant difference is observed between synthetic and biological mesh in mesh-related complications [85, 87–89]. Evidence suggests, however, that recurrence rates are higher following a repair with biological mesh compared with synthetic mesh [6, 29, 89, 90]. To reduce the risk of mesh erosion, we administered preoperative antibiotics, dissected meticulously with strict monitoring of hemostasis to prevent a hematoma, attached the (type I) mesh, and closed the incised peritoneum over the mesh. But considering the numerous risk factors and prevention strategies, the occurrence of mesh erosion presumably has a multifactorial origin. Mesh erosion after laparoscopic ventral rectopexy has been described to occur in the rectum, vagina or bladder, and strictures or rectovaginal fistulas have also been described [91]. In this study, we have not found rectal mesh erosion, nor did we have patients with symptoms suggesting fistulas or strictures.

Four (4.2%) patients in this study experienced vaginal discomfort during speculum examination. Two of these 4 patients (both sexually active) occasionally experienced vaginal discomfort in daily life. The possibility of vaginal discomfort, probably due to vaginal atrophy and reduced elasticity of the vaginal wall caused by the mesh, should be considered in the decision to offer pelvic reconstructive surgery using mesh in older sexually active females. Both the rectum and the vagina were examined in this study, but only vaginal erosions were diagnosed. The most probable explanation for this difference is vaginal atrophy, which increases with age. In order to obviate this, surgeons could consider prescribing vaginal estrogen cream pre- and postoperatively.

The erosion rates in the literature are in line with our erosion rates. However, the majority of the studies in the literature were retrospective and lacked a systematic follow-up with a rectal and vaginal examination. Furthermore, this study proves that mesh erosion can also occur asymptomatically. The clinical significance of an asymptomatic mesh erosion is, however, unclear. Only the patient with symptomatic mesh erosion underwent surgical intervention in our series. Because of the difference in methods and follow-up, the retrospective design and the lack of mentioning asymptomatic erosions, it is likely that erosion rates are underestimated in the current literature. We believe that the erosion rate in this study approaches the true rate.

The strong points of this study were its prospective nature, with the use of validated questionnaires and standardized follow-up examinations to confirm our findings. Loss to follow-up was low considering the long duration of the study and reasons for loss to follow-up were known. Furthermore, solely type 1 mesh was used throughout this study, minimizing heterogeneity and variability. Another strong point is that it reports not only on sacrocolpopexy, but also on combined sacrocolporectopexy, making the results more widely applicable.

The most important limitation of this study is that all patients were treated in a single tertiary referral hospital for pelvic floor disorders. Some of the patients had complex pelvic floor disorders and/or an extensive history of pelvic floor surgery, therefore limiting the generalizability of the results. In addition, 26.1% of all invited patients were not physically examined for various reasons, and therefore bias may have occurred. These patients were, however, assessed using a questionnaire specifically assessing erosion-related complaints. We aimed for a 5-year follow-up; however, the follow-up time ended up being 48.1 months. Most patients were examined between 43 and 54 months (interquartile range), therefore limiting our Kaplan–Meier estimates at the exact time point of 60 months. We added 95% confidence intervals to make our results more accurate and interpretable with the wider range of follow-up. Results of the Kaplan-Meier curve should therefore be interpret with caution. Another limitation is that we did not perform a power analysis. This study was set up as an observational cohort study, and our hypothesis, based on literature, was to find a low incidence, and significant prognostic factors were therefore not expected. In our literature review, studies with different inclusion criteria and methods were included. This impaired the homogeneity of the literature results.

Mesh-related morbidity is an important issue because of the potential impact on the quality of life, the widespread use of mesh and the global attention to the topic. In recent years, the public opinion has turned fiercely against the use of synthetic grafts. Fear of mesh-related morbidity is resulting in under-treatment of all serious, disabling pelvic floor disorders. The results of this study and the literature review demonstrate that abdominally placed synthetic meshes for pelvic reconstructive surgery has a low complication rate in the long-term. This is an encouraging finding for patients, doctors, and governmental institutions, in a field marked by a lack of knowledge about the use of mesh. Surgeons using synthetic mesh for pelvic floor repair are encouraged to perform focused and meticulous examinations looking for mesh erosion in the long-term to confirm these results.

## Electronic supplementary material

ESM 1 (DOCX 63 kb)
