# Supplementary material for: Long-term mesh erosion rate following abdominal robotic reconstructive pelvic floor surgery: a prospective study and overview of the literature
Source: Int Urogynecol J. 2019 Jun 20;31(7):1423–33. doi: 10.1007/s00192-019-03990-1 (PMC7306026; doi:10.1007/s00192-019-03990-1)

**Appendix A. An overview of the literature.**

A computerized search in Pubmed/Medline for English-language articles until January 31th, 2017 was performed, using the terms: ‘sacrocolpopex*’ OR ‘sacral colpopex*’ OR ‘sacrocervicopex*’ OR ‘cervicopex*’ OR ‘colpopex*’ OR ‘(rectopex* AND sacr*)’ OR ‘(rectopex* AND colp*)’ OR ‘sacrocolporectopex*’ OR ‘colporectopex*’ OR ‘rectovaginopex*’ OR ‘sacrocolpoperineopex*’ OR ‘colpoperineopex*’. Studies describing mesh erosion after minimal invasive sacrocolpopexy with a minimum follow-up of 12 months were included (Figure 3). Titles and abstracts were scrutinized by two researchers independently (FZ,JI). Full manuscripts of all citations that were likely to meet the predefined selection criteria were obtained. References of obtained articles were scanned to identify other potentially eligible articles. Articles describing solely on non-synthetic types of mesh and pediatric articles were excluded. Studies describing mesh erosion after sacrocolporectopexy were described separately. Both open and minimal invasive procedures were included, as the number of studies describing mesh erosion after the combined approach were limited. No separate distinction in duration of follow-up was made.

**Appendix A. Figure 3. Flowchart of included studies.**

Legend:

Abbreviations: SC: sacrocolpopexy; LSC: laparoscopic sacrocolpopexy; RSC: robot-assisted laparoscopic sacrocolpopexy.


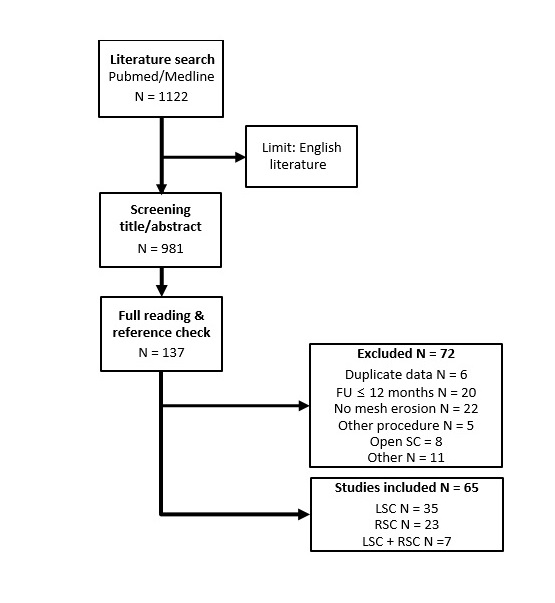

Supplement: Supplementary file 1 — (DOCX 63 kb) [file 192_2019_3990_MOESM1_ESM.docx]
